# Pre-Exercise Whole- or Partial-Body Cryotherapy Exposure to Improve Physical Performance: A Systematic Review

**DOI:** 10.3390/sports9100135

**Published:** 2021-09-30

**Authors:** Emily M. Partridge, Julie Cooke, Andrew J. McKune, David B. Pyne

**Affiliations:** 1 Research Institute for Sport and Exercise Science (UCRISE), University of Canberra, Bruce, ACT 2617, Australia; julie.cooke@canberra.edu.au (J.C.); Andrew.McKune@canberra.edu.au (A.J.M.); David.Pyne@canberra.edu.au (D.B.P.); 2 Faculty of Health, University of Canberra, Bruce, ACT 2617, Australia; 3 Discipline of Biokinetics, Exercise and Leisure Sciences, School of Health Sciences, University of KwaZulu-Natal, Durban 4041, South Africa

**Keywords:** cooling, physical performance, biomarkers, neuromuscular facilitation

## Abstract

Whole- (WBC) and partial-body cryotherapy (PBC) are commonly used sports medicine modalities for the treatment of injury and exercise recovery. Physiological and perceptual effects have the potential to be utilised in a novel application that involves pre-exercise WBC and PBC exposure to improve physical performance. A systematic literature search of multiple databases was conducted in July 2021 to identify and evaluate the effects of pre-exercise exposure of WBC or PBC on physical performance measures, and any potential translational effects. The following inclusion criteria were applied: (1) use of WBC or PBC exposure pre-exercise, (2) use of WBC or PBC in healthy and/or athletic populations, (3) control group was used in the data collection, and (4) investigated physiological, psychosocial or direct physical performance impacts of pre-exercise cryotherapy exposure. A total of 759 titles were identified, with twelve relevant studies satisfying the inclusion criteria after full-text screening. The twelve studies were categorised into three key areas: performance testing (n = 6), oxidative stress response (n = 4) and lysosomal enzyme activity (n = 2). The potential for eliciting favourable physical and physiological responses from pre-exercise WBC or PBC is currently unclear with a paucity of good quality research available. Furthermore, a lack of standardisation of cryotherapy protocols is a current challenge.

## 1. Introduction

The hours leading up to competition are a critical window for preparing individual and team athletes both psychologically and physically to achieve peak performance. This window of preparation typically involves active and passive methods with the goals of enhancing oxygen uptake, cardiac output, blood flow to skeletal muscle, neuromuscular activation and mental alertness. While these methods are generally prioritised by individuals or within teams, performance enhancement activities are often based on personal preferences prioritised by coaches and athletes. With technology advancing, more diverse, extreme and novel preparation tactics are being employed to enhance the readiness and overall physiological state of athletes.

Whole- (WBC) and partial-body cryotherapy (PBC) are sports medicine modalities that expose users to air temperatures of −10 °C to −170 °C for periods between 20 s to 3 min [1]. PBC was the first form of cryotherapy to use nitrogen gas and developed to alleviate pain and treat rheumatic disease, neurodegenerative disorders and inflammatory conditions [2]. Forms of cryotherapy that have been utilised include exposure to cold air or water [3]. The temperature achieved has generally been limited by the ~40-fold lower thermal conductivity of air (0.0151 W·mK^−1^) compared with water (0.5846 W·mK^−1^) [4]. This marked difference in thermal conductivity permits much colder temperatures to be reached using air (rather than cold water immersion) while maintaining a high level of safety for the user [5]. The two cryotherapies also differ in the type of chamber utilised, protocols employed, and temperatures of the chamber/sauna. PBC involves individuals being exposed to vaporised liquid nitrogen in a single user, head-free cryochamber. Protocols usually involve 2–3 min of exposure at −110 °C to −170 °C [6]. WBC is a variant of this, typically involving a multi-user cryosauna with a closed multi-chamber system [6]. Protocols for WBC usually involve 20–30 s exposure in temperatures of −10℃ to −60 °C, then 2 to 3 min in −110 °C to −150 °C [7]. Despite the extensive use of both cryotherapies, the widely varying protocols make drawing direct conclusions from the available evidence difficult.

Both forms of cryotherapies have more recently gained popularity as a means of promoting well-being and recovery for athletes [8] and are primarily employed to promote recovery after exercise-induced muscle damage [9]. WBC exposure after simulated trail running in athletes elicited reductions in perceived sensations of muscle pain and restored maximal muscle strength 1 h post-exposure better than 24 h of passive recovery [10]. However, cold-water immersion post-resistance exercise by recreationally active males yielded a reduction of myofibrillar protein synthesis rates which could impair muscle conditioning [11]. The evidence is lacking in regard to cold therapies post-exercise improving muscle recovery from resistance or aerobic exercise and is not fully understood.

Direct physiological effects from the use of post-exercise cryotherapy have been reported, including substantial increases in testosterone [12], blood catecholamines (norepinephrine, epinephrine and dopamine) [13], salivary α-amylase (a biomarker of autonomic nervous system activation) [14], decreases in markers of oxidative stress (lipid peroxidation and antioxidants) [15,16] and anti-inflammatory biomarkers (e.g., IL-1β, IL-6, protein C-reactive (CRP) and tumor necrosis factor alpha (TNF-α)) [17]. WBC exposure also influences heart rate variability (HRV) measures [18] and decreased perceived muscle fatigue [3]. WBC and PBC purportedly enhances cardiovascular fitness, sleep quality, temperature regulation, and increases energy intake, appetite regulation, overall mood and well-being [19,20,21]. With traditional cryotherapy exposure limited to post-exercise recovery, we recently proposed that translating the physiological effects into a novel pre-exercise cryotherapy exposure holds promise for improving subsequent physical performance [22]. WBC and PBC could elicit a significant pre-exercise potentiation effect (analogous to the classical priming technique of post-activation potentiation (PAP)) to enhance subsequent exercise or sporting performance. The desired outcome is to enable athletes to perform at a higher level for both training, competition and to maintain performance over long training periods by potentially limiting or avoiding illness or injury.

The objective of this review was to identify and evaluate studies examining the effects of pre-exercise WBC or PBC on physical performance and the physiological effects following exposure and exercise. WBC and PBC exposure have been extensively researched for post-exercise muscle recovery effects. However, the application of these modalities’ pre-exercise and the potential beneficial physiological and physical effects for athletes are yet to be determined. Establishing a theoretical approach is needed to evaluate potential practical applications of a cryotherapy exposure in an athletic setting. This review is needed to clarify whether pre-exercise cryotherapy exposure offers worthwhile benefits for enhancing athletic performance for both the pre-competition preparation period and targeted training sessions.

## 2. Methods

### 2.1. Search Strategy

**Figure 1:**
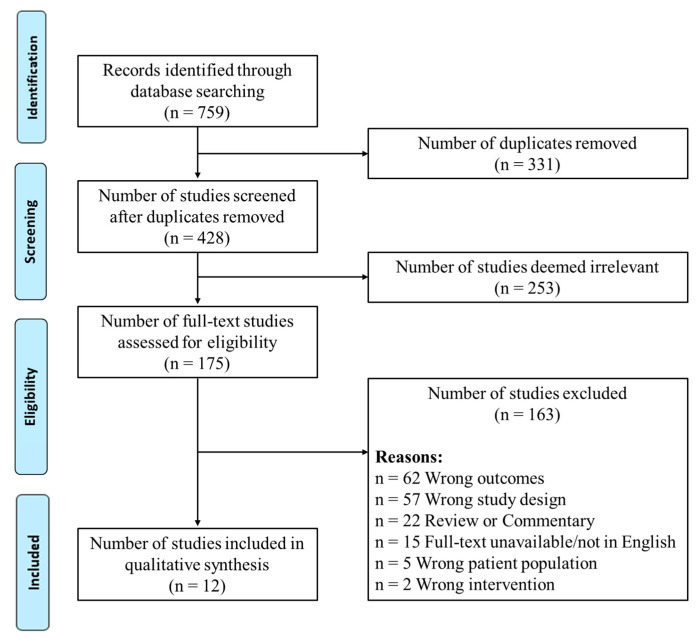
PRISMA flow chart, PRISMA: Preferred Reporting Items for Systematic Reviews and Meta-Analysis.

This systematic review was conducted in accordance with the guidelines from the preferred reporting items for systematic reviews and meta-analysis (PRISMA) [23,24]. The search strategy is outlined in Figure 1.

### 2.2. Literature Search Methodology

A comprehensive electronic search was conducted in July 2021 using SportDiscus, PLOS One, Web of Science and PubMed databases with limitations of English language and articles from 2000 to July 2021. All potential references were imported into Endnote X9 (Thompson Reuters, Carlsbad, CA, USA), then into Covidence (Covidence systematic review software, Veritas Health Innovation, Melbourne, Australia) and 331 duplicates were removed.

### 2.3. Search Parameters and Criteria

The following inclusion criteria were applied: (1) use of WBC or PBC exposure pre-exercise, (2) use of WBC or PBC in healthy and/or athletic populations, (3) control group was used in the data collection, and (4) investigated physiological, psychosocial or direct physical performance impacts of pre-exercise cryotherapy exposure. The initial search keywords were “whole-body cryotherapy”, “whole-body cryostimulation”, “partial-body cryotherapy” or “partial-body cryostimulation”. The terms “cryotherapy” and “cryostimulation” were both included as the terms are often used interchangeably when referring to a cryochamber or cryosauna. Two authors (E.M.P., J.C.) separately and independently reviewed search returns for eligibility. Any discrepancies between the two reviewers were discussed until a consensus was reached, with unsettled differences resolved in consultation with a third author (A.J.M.). During full-text screening, the two most common reasons for exclusion were: (1) the study design involved WBC or PBC used post-exercise for recovery purposes, and (2) no exercise was used in the protocol. Specifically, the excluded studies had a focus on physiological responses, e.g., skin temperature, heart rate or blood pressure with no physical activity involved pre-exposure.

### 2.4. Data Extraction

Data were extracted by the lead author (E.M.P.) and included participant characteristics (e.g., sample size, gender, age, height, mass), study characteristics (e.g., cryotherapy modality, protocol, duration from cryotherapy stimulus and performance or physiological outcome measures). After extraction, the information was reviewed in consultation with a second author (J.C.).

### 2.5. Assessment of Study Quality

The methodological quality of each study was assessed by two investigators (E.M.P. and J.C.) using the Black and Downs checklist tool [25], and scores examined to establish consensus between assessors. Each study was assessed on items in the following categories: (1) reporting, (2) external validity, (3) internal validity (bias), (4) internal validity (confounding—selection bias), and (5) statistical power. The quality of the studies was determined by a point accumulation (total points 28) with 26–28 rated as excellent, 20–25 good, 15–19 fair and <14 poor quality [26].

## 3. Results

The electronic search identified 759 records; duplicates were then removed, leaving 428 studies. The titles and abstracts were screened, which reduced the number of studies to 175 for full-text review. The remaining full texts were obtained and assessed for eligibility against the inclusion criteria. A total of 163 articles were excluded for not meeting the inclusion criteria. The two most common exclusion reasons were: (1) the study design involved WBC or PBC post-exercise for recovery purposes, and (2) no routine training or physical testing was used in the study protocol (i.e., post-exposure). The remaining twelve studies were included for full review and analysis. This process is presented in Figure 1. The characteristics of the twelve studies included are presented in Table 1 detailing the participant characteristics, intervention and control protocols, and results of each investigated variable.

The twelve identified studies were categorised into 3 key research areas: physical performance (n = 6) [27,28,29,30,31,32], oxidative stress response (n = 4) [33,34,35,36] and lysosomal enzyme activity (n = 2) [37,38]. Within these categories, three studies used WBC exposure acutely prior to testing or physiological measures being taken [27,29], and five utilised WBC chronically (2–3 exposures per day over a 6–10 training period) [33,34,35,37,38]. The remaining four studies utilised PBC exposure acutely prior to physical performance measures [28,32,36], with one study misclassifying the cryotherapy modality as WBC, when the correct term is PBC, given the participants’ head was out of the chamber [30].

**Table 1:** Summaries of WBC and exercise studies by intervention type, protocol, description of study participants and results.

Intervention Type and Protocol							
Authors [Reference No.]	Participants (Male/Female)	Age (Age ± SD Years)	WBC or PBC	Intervention Group	Control Group	Exposure and Period of Time	Results
Costello et al. (2012) [29]	12 healthy men and 6 healthy women in experimental and control group	20.8 ± 1.2	WBC	20 s at −60 °C then 3 min in −110 °C	Both chambers set to 15 ± 3 °C	Two exposures with 2 h between each exposure	WBC had no effect on maximal voluntary isometric contraction and peak power output of the knee extensors.
De Nardi et al. (2020) [31]	11 healthy women	28.0 ± 2.2	PBC	2.5 min at −130 °C to −170 °C	2.5 min in a thermoneutral environment (22.0 ± 0.5 °C)	One exposure prior to same day testing	PBC exposure improved sit and reach flexibility scores (+2.8 cm) compared to control (+1 cm). Active knee extension was improved range of motion more after PBC (+11.8%) than control (−0.7%).
De Nardi et al. (2017) [28]	50 healthy men and 50 heathy women	Men: 42.4 ± 14.2 Women: 37.1 ± 9.3	PBC	2.5 min at −130 °C to −160 °C	2.5 min in a thermoneutral environment (22.0 ± 0.5 °C)	One exposure prior to same day testing	PBC exposure improved grip strength in women after (+2.0 kg) compared with control (+0.5 kg) and in men (+1.5 kg) compared to the control (+0.5 kg).
De Nardi et al. (2015) [27]	60 healthy men and 60 healthy women	Men: 33.7 ± 9.9 Women: 32.8 ± 10.4	WBC	2.5 min at −130 °C to −140 °C	2.5 min in a thermoneutral environment (22.0 ± 0.5 °C)	One exposure prior to same day testing	Increase in sit-and-reach score in men after WBC (+2.3 cm) compared to control (+0.2 cm) and in women in both WBC and control group (+3.1 cm and +0.8 cm, respectively).
Ferreira-Junior et al. (2014) [30]	13 healthy, physically active men	27.9 ± 4.2	PBC *	3 min at −110 °C	3 min in a thermoneutral environment (21 °C)	One exposure prior to same day testing	No significant difference from baseline for peak torque between WBC (+1.6 N.m) and control condition (+1.5 N.m). Unclear power output changes for both WBC and control (0.8 W and 0.8 W, respectively).
Mila-Kierzenkowska et al. (2013) [36]	18 professional male volleyball athletes	28.3 ± 4.0	WBC	10–20 s at −10 °C then 2 min at −130 °C	No exposure to cryogenic temperatures	One exposure prior to same day testing	CAT was two times higher pre-post in control condition (+10%) and two times lower in WBC condition (−0.5%). IL-6 was significantly lower after WBC (−14%) exposure compared to control (+124%).
Mila-Kierzenkowska et al. (2011) [38]	Nine Olympic team female kayakers	23.9 ± 3.2	WBC	3 min at −120 °C to −140 °C	No exposure to cryogenic temperatures	Twice a day exposure over a 10-day training cycle	Decreased concentrations of ASA (43%), AcP (40%) after 6 days of cryostimulation.
Mila-Kierzenkowska et al. (2009) [33]	Nine professional female kayakers	23.9 ± 3.2	WBC	3 min at −120 °C to −140 °C	No exposure to cryogenic temperatures	Twice a day exposure over a 10-day training cycle	Decreased concentration of SOD (−10%), CAT (−21%), TBARS were significantly reduced at both 6-days post-WBC exposure (−34%).
Partridge et al. (2021) [32]	Twelve male rugby union athletes		PBC	3 min at −140 °C	No exposure to cryogenic temperatures	One exposure 3 h before same day testing	Improved CMJ peak velocity (+4.9%), increase in sAA concentration (+131%) 15 min post-PBC, and improvements in perceived muscle soreness, mood and mental fatigue.
Wozniak et al. (2013) [34]	Six elite male rowers	26.7 ± 3.6	WBC	3 min at −125 °C to −150 °C	No exposure to cryogenic temperatures prior to training	Twice a day exposure over a 6-day training cycle	Decrease in SOD (−44%), CD (−35%), GPx (−42%), and AcP (−50%) after 3-days of daily WBC exposure. TBARS concentration after 6 days of exposure decreased 50%.
Wozniak et al. (2007) [37]	20 Olympic team male kayakers and 10 untrained men	Athletes: 24.8 ± 4.1 Untrained: 26.9 ± 4.1	WBC	3 min at −120 °C to −140 °C	No exposure to cryogenic temperatures prior to training	Twice a day exposure over a 10-day training cycle	Decrease in CD in plasma by −46%, TBARS −24%, SOD −47% lower, and GPx −50% after 6 days of WBC exposure.
Wozniak et al. (2007) [35]	21 Olympic team male kayakers and 10 untrained men	Athletes: 24.6 ± 4.3 Untrained: 26.9 ± 4.1	WBC	3 min at −120 °C to −140 °C	No exposure to cryogenic temperatures prior to training	Three WBC exposures a day over a 10-day training cycle for athlete group and one WBC exposure a day for untrained men.	Decreased concentrations of ASA (46%), AcP (32%) and CK (34%) with WBC exposure 10-days of regular training.
WBC: whole-body cryotherapy PBC: partial-body cryotherapy CAT: catalase IL-6: interleukin 6 ASA: arylsulfatase CK: creatine kinase sAA: salivary α-amylase					AcP: acid phosphatase SOD: superoxide dismutase TBARS: thiobarbituric acid reactive substance CD: conjugated dienes GPx: glutathione peroxidase CMJ: countermovement jump		

* The cryotherapy modality was misclassified as whole-body cryotherapy when the study actually used partial-body cryotherapy, distinguished by the participants head being out the chamber.

Seven of the twelve included studies examined an elite athlete cohort [33,34,35,36,37,38]. Four of these used Olympic team kayakers [33,35,37,38], one had elite rowers [34], one used professional volleyball athletes [36] and the last used semi-professional rugby union athletes [32]. Most of these studies investigated oxidative stress, and lysosomal enzyme activity with only one study investigating physical performance measures [32]. The five remaining studies used healthy, recreationally active participants to investigate the neuromuscular facilitation response to pre-exercise WBC or PBC exposure [27,28,29,30].

There was marked variability in WBC and PBC protocols used for each of the twelve studies. Four WBC studies used an exposure of 3 min at −120 °C to −140 °C [33,35,37,38], with the remaining four studies using all different exposures including 3 min at −125 to −150 °C [34], 2.5 min at −130 to 140 °C [27], 20-sec acclimation at −60 °C then 3 min at −110 °C, and finally a protocol of 10–20 sec acclimation at −10 °C then 2 min at −130 °C [36]. The three studies using the PBC intervention featured varying protocols of time and temperature exposure including 2.5 min at −130 °C to −170 °C [31], 2.5 min at −130 °C to −160 °C [28], 3 min at −110 °C [30] and 3 min at −140 °C [32]. Only five studies described the control group intervention, where the participants spent an equal time to the WBC exposure in a thermoneutral environment. Of the other six studies, no indication of the environmental conditions prior to testing or samples being drawn was specified for the control group.

The risk of bias and quality assessment indicated that no studies were of excellent quality, four were good quality (33%), six were fair quality (50%), and two were deemed of poor quality (17%). A significant contributor to these assessments was the lack of blinding in all studies, unclear statistical analysis processes, or a power analysis was not indicated. The mean score of the 12 studies was of fair quality. The risk of bias assessments and scores are detailed in Table 2.

Acute PBC (3 min at −140 °C) exposure 3 h before countermovement jump testing, using GymAware software, in male rugby union athletes elicits improvements in peak jump velocity by 4.7 ± 3.5% compared to a −1.9 ± 4.8% decrease in the control condition [32]. Alongside the physical performance, this study also quantified a +131% increase in salivary α-amylase enzyme concentration compared to a −4.2% decrease in control and improvements in self-reported readiness to perform variables of mental fatigue, mood and muscle soreness. Acute WBC exposure prior to a sit-and-reach test in healthy, middle-aged participants elicited a significant increase in reach amplitude in men by ~2.3 ± 1.4 cm compared with the control condition, and an improvement by ~2.4 cm ± 1.6 in women [27]. Similarly, PBC exposure improved the sit-and-reach score by +1.8 ± 0.9 cm more compared to the control condition and increased ROM during active knee extensions by +11 ± 3.9% change compared with a decrease of −0.7 ± 0.5% change in the control group [31]. Isometric grip strength was evaluated pre and post-acute PBC exposure in healthy men and women. A larger increase in grip strength was seen in women after PBC exposure (+1.4 ± 0.4 kg) compared to the control condition. Similarly, a larger increase in grip strength was seen in men post-PBC exposure (+1.0 ± 0.8 kg) compared to the control condition [28]. In another study investigating acute WBC exposure, there was minimal difference in the maximal isometric contraction of the knee extensors between groups. Furthermore, peak power output was also unaffected by the WBC treatment [29]. The last neuromuscular facilitation and performance study utilised healthy, recreationally active men and investigated the effects of acute PBC exposure on maximal isometric elbow flexion torque. Peak torque change from baseline indicated no difference between the WBC and control conditions (+0.1 ± 1.2 N·m). Similar results were seen in the mean power output between-group difference scores (0.0 ± 0.1 W) with unclear changes [30].

There were favourable results in the two studies that investigated lysosomal enzymatic activity in elite athlete cohorts. After six days of cryostimulation, compared with the control condition, blood serum concentrations of arylsulfatase (ASA) and acid phosphatase (AcP) decreased by 43–46% and 27–40% respectively [37,38]. Creatine kinase (CK) increased by +92% compared to a +193% increase in the control condition [37]. Training preceded by cryotherapy can stabilise lysosomal membranes and potentially reduce micro-injury to muscle fibres caused by intense exercise.

The four studies investigating the oxidative stress response to exercise following WBC exposure, measured the intrinsic antioxidants, superoxide dismutase (SOD), catalase (CAT), and glutathione peroxidase (GPx). Markers of lipid peroxidation, which indicate oxidative stress in an organism, thiobarbituric acid reactive substance (TBARS) and conjugated dienes (CD) were also measured. The studies also investigated creatine kinase (CK) as an indirect marker of muscle damage and the inflammatory biomarker, interleukin-6 (IL-6). In the study using elite volleyball athletes, CAT was 10% higher pre-post in the control condition and 0.5% lower in WBC exposure group [36]. IL-6 was also significantly lower by 14% after WBC exposure compared to an increase of 124% in the control condition [36]. This result indicates less oxidative stress in the group using WBC given the lower levels of inflammation and antioxidant markers. In elite male rowers, favourable decreases in SOD, GPx antioxidants and the lipid peroxidation markers of CD and TBARS were recorded after 3 days of twice-daily WBC exposure compared to an increase in all variables in the control group [34]. The favourable decreases in lipid peroxidation markers, and therefore oxidative stress from WBC exposure, results in a lower need for antioxidants; hence, the greater increase of antioxidants in the control condition. In the two studies using elite kayakers, similar results were found, with both studies reporting a significant decrease from baseline in the antioxidants GPx (−68%) and CAT (−21%) after 10 days of WBC exposure indicating a reduced oxidative stress response from exercise [33,35]. Significant decreases in concentrations at 6 days and 10 days of WBC exposure were observed in the antioxidant SOD by 36–47% and oxidative stress indicator TBARS by 21–34% [33,35]. In summary, it appears that cryotherapy exposure pre-exercise can elicit significant favourable decreases in markers of oxidative stress, and lower the subsequent response of antioxidants to exercise stress.

## 4. Discussion

Elite athletes are typically in an ongoing state of recovery management, given the frequency of high-volume and high-intensity training sessions. This is especially true for sports that have weekly competitions, multi-day tournaments, or multiple competitions over a short period. To date, WBC and PBC exposure has mostly been explored in a post-exercise application. However, the potential of both enhancing performance and recovery using WBC or PBC prior to competition or high-intensity exercise, is of interest to coaches and players and warrants further investigation. This systematic review provides preliminary evidence supporting the acute and chronic use of PBC and WBC pre-exercise to enhance subsequent performance in healthy individuals and athletes. Cryotherapy has been shown to enhance neuromuscular facilitation, decrease lysosomal enzymatic activity, oxidative stress and inflammatory markers. However, the majority of studies were rated of only poor to fair quality given a lack of blinding, and shortcomings in the statistical analysis and reporting of results.

In this review, the five of the six studies investigating physical performance used healthy, recreationally active participants and not an athletic population. PBC has induced increases in sit-and-reach performance by up to 3 cm in both healthy men and women [31]. There was a similarly favourable neuromuscular facilitation response using sit-and-reach amplitude after WBC exposure [27], that could translate into other physical performances or fitness monitoring tests. This effect could relate to an analgesic effect of perceived pain reduction concerning range of motion limits. In elite athletes, analgesic effects can be beneficial for performance outcomes, however, a reduction in perceived pain could paradoxically increase the risk of an injury due to a dampened pain response to exercise loads that might exceed a critical threshold or tolerance limit. Despite this, the application of wet-ice application and cold-water immersion immediately prior to physical performance testing has previously demonstrated no adverse effects in physically active participants [39].

Pre-cooling methods have been used extensively by athletes prior to being exposed to hot environments using internal (cold fluid ingestion) or external cooling (ice vests, packs or cold-water immersion). These methods have yielded improvements in both anaerobic and aerobic exercise performance [40]. However, the direct relationships between cryotherapy and performance in an athletic population have not been directly studied. Peak torque during isometric elbow flexion [30] and knee extension [29] was unchanged in recreationally active participants after cryotherapy exposure. A greater increase in grip strength was observed in both men and women [28], indicating that a pre-cooling method of cryotherapy exposure can increase selected strength-based performances. Furthermore, the performance testing was completed immediately after cryotherapy exposure in all studies, so the longevity of these effects is unknown which is an important variable for the realistic application of cryotherapy in an athletes’ preparation routine.

Seven of the included studies examined the effects of WBC or PBC in an elite athletic cohort. However, six studies did not determine the direct impacts of cryotherapy exposure on sport-specific performance measures, focusing instead on physiological biomarkers. One area of investigation was reactive oxygen species (ROS), which are formed through physical exercise and counteracted by nutritional antioxidants and the endogenous enzymatic antioxidant system [41]. An imbalance between ROS and antioxidants can lead to oxidative stress in individuals [42]. Regular, intense training and competition, without adequate recovery or reduced training load, can increase the level of oxidative stress markers that contributes to increased risk of illness and injuries in athletes [43]. An increase in the oxidative stress index (OSI) in elite Olympic rowers was related to higher injury and illness rates during the competition phase of their season [44]. The marked decreases in ASA, AcP and CK facilitated by WBC or PBC could potentially decrease injury and illness risks over long, intense training periods when used chronically by athletes.

Four of the included studies investigating exercise-induced oxidative stress responses reported a substantial decrease in markers of oxidative stress after pre-exercise WBC or PBC exposure conditions compared to a control condition. Malondialdehyde (a marker of lipid peroxidation) in elite cyclists over a multi-day tournament was elevated by ~50% after the final stage of racing compared to the end of the first stage [45]. This outcome indicates the presence of an inflammatory state as a consequence of the repetitive, intense exercise over a short period. The favourable decrease in oxidative stress variables indicated by WBC or PBC exposure could shorten recovery periods after high-intensity training sessions, or for sports involving weekly competitions or multi-day tournaments. However, the heightened oxidative stress from exercise is important for adaptation and muscle regeneration from training [46]. To promote this adaptive state, it is vital for an athlete’s training load to balance positive physiological adaptations with the underlying oxidative stress state [47]. The relationship between the dampening of the oxidative stress response to exercise from cryotherapy exposure and the subsequent performance by athletes is yet to be fully understood.

Intense exercise is likely one explanation for the increased imbalance in homeostasis [38]. Lysosomes are the organelles responsible for breaking down complex chemicals and play a crucial role in cellular repair from muscular injury caused by exercise [48]. The increased release of lysosomal hydrolases into the bloodstream can challenge cellular repair from injury [49]. A substantial decrease in the concentrations of AcP and ASA in participants exposed to WBC prior to exercise could shorten recovery periods between training and reduce muscle cell inflammation in individuals after intense training. This response was noted after chronic use of WBC over 6- and 10-day periods, and it is unknown whether similar decreases in lysosomal concentration also occurs after acute cryotherapy exposure. There is a clear need for further evidence regarding acute and chronic exposures of cryotherapy to clarify the protocol(s) needed for favourable changes in enzyme concentrations.

The two cryotherapy modes of WBC and PBC currently used by athletes’ lack standardization and are subject to the preferences of the sport practitioners or private companies applying the therapeutic modality. The variations of cryotherapy mode, temperature, and duration of exposure in the different chambers make the investigation of critical factors problematic. Few studies have directly compared the two cryotherapy modes with a control to identify marked differences on physiology and subsequent performance benefits. WBC can elicit a greater decrease in tympanic temperature, blood pressure, increased sensations of user discomfort, and larger stimulation of the autonomic nervous system than PBC exposure [13]. WBC likely elicits a greater reduction in tympanic temperature, meaning the more a body is cooled, the greater response independent of physiological measures. Any pronounced differences or similarities between the two cryotherapy modes are mostly unknown given methodological variability in protocols used by either mode complicating the issue.

### 4.1. Quality and Risk of Bias

While there was a high risk of bias and low quality in the identified studies, we acknowledge it is difficult to blind the intervention of WBC or PBC to study participants (and the research team) potentially leading to bias and misclassification of effects. Single blinded studies are possible by using higher temperatures for the WBC or PBC exposure to elicit a degree of blinding and strengthen studies [29]. However, future studies investigating both physical and mental preparation or performance should implement a degree of blinding for the participants to counter any subjective bias in findings. Furthermore, the reporting standards were lacking in quality for most of the studies extracted. Specifically, the lack of contraindications considered when using cryotherapy were not detailed despite many known to impact participants health. There was little reporting of external confounding factors, specifically within the training cycles of the elite athlete cohorts. No study reported any prior power analyses to verify whether the cohort sample size was adequate.

### 4.2. Future Research

Athletes can specialise in a broad spectrum of events, playing positions, and sports with various physiological demands, and this could dictate the influence cryotherapy exposure has on aspects of athletic performance. The distinct lack of evidence-based research limits the understanding of coaches and sport practitioners on the potential novel applications of WBC or PBC exposure for athletic groups. For a more informed approach to sport-specific use of pre-exercise cryotherapy exposure, further research should investigate the influence of passive pre-activation or priming in conjunction with routine dynamic warmups. Additionally, more sport-specific testing, cross-sectional and prospective longitudinal research is needed in various elite athlete cohorts to understand the impact on complex variables inherent within this population. Furthermore, the effect of training type, dosage (intensity and duration) on the subsequent effects of pre-exercise WBC or PBC exposure could have underlining effects on these findings and should be explored more in elite athletes. From this review, it can be seen that there is a paucity of evidence using sport-specific testing of performance measures using athletes when using WBC or PBC pre-exercise or pre-competition, such as sprints, repeated sprints or simulated gameplay and racing.

## 5. Conclusions

The application of WBC and PBC for athletes in the preparation period (hours) immediately prior to training or competition has the potential for enhancing performance. Few studies have addressed the acute effects of pre-exercise cryotherapy exposure on subsequent physical performance outcomes. The effects of a dampened WBC or PBC induced oxidative stress response, lysosomal enzymatic activity, and enhanced neuromuscular facilitation of physical performance, are yet to be fully described with quality evidence-based research. Other physiological and psychosocial factors (e.g., hormonal concentrations and variables of mental well-being or competition readiness) of performance identified in selected studies of WBC and PBC exposure have not been investigated thoroughly and provide a large area of opportunity. Coaches, sport scientists and sports medicine practitioners are expressing interest in new methods for preparing athletes for competition with simple and effective interventions to promote performance. WBC and PBC could provide valuable physiological and performance benefits for athletes in both training and competition, but applications are currently limited by non-uniform protocols of cryotherapy use. 

## Figures and Tables

**Table 2:** Quality assessment scores based on the Black and Downs checklist.

Study (Year Published)	Reporting	External Validity	Internal Validity (Bias)	Internal Validity (Confounding)	Power	Total Score		Quality of Study
						Total (28)	%	
Costello et al. (2012)	8	3	5	4	0	20	71	Good
De Nardi et al. (2020)	7	3	5	4	1	20	71	Good
De Nardi et al. (2017)	7	3	4	4	0	18	64	Fair
De Nardi et al. (2015)	7	2	5	4	0	18	64	Fair
Ferreira-Junior et al. (2014)	9	2	5	3	1	20	71	Good
Mila-Kierzenkowska et al. (2013)	7	3	5	4	0	19	67	Fair
Mila-Kierzenkowska et al. (2011)	6	2	4	2	0	14	50	Poor
Mila-Kierzenkowska et al. (2009)	5	2	4	3	0	14	50	Poor
Partridge et al. (2021)	9	3	5	5	1	23	82	Good
Wozniak et al. (2013)	6	3	4	3	0	16	57	Fair
Wozniak et al. (2007)	5	2	4	4	0	15	53	Fair
Wozniak et al. (2007)	6	2	4	4	0	16	57	Fair
Total for studies	82	30	54	44	3			
% for studies	68.3	83.3	64.3	61.1	25.0			
Average	6.8	2.5	4.5	3.7	0.3	17.8	63.1	Fair

All criteria were scored on the following scale: Yes = 1; No = 0 and Unable to Determine = 0.

## Data Availability

The data presented in this study are available on request from the corresponding author.
